# Predictors of metabolic-associated fatty liver disease (MAFLD) in adults: a population-based study in Northeastern Iran 

**Published:** 2021

**Authors:** Ehsaneh Taheri, Alireza Moslem, Alireza Mousavi-Jarrahi, Behzad Hatami, Mohammad Amin Pourhoseingholi, Hamid Asadzadeh Aghdaei, Mohammad Reza Zali

**Affiliations:** 1 *Gastroenterology and Liver Diseases Research Center, Research Institute for Gastroenterology and Liver Diseases, Shahid Beheshti University of Medical Science, Tehran, Iran*; 2 * Cellular and Molecular Research Center, Sabzevar University of Medical Science, Sabzevar, Iran*; 3 *Department of Community Medicine, Faculty of Medicine, Shahid Beheshti University of Medical Science, Tehran, Iran*; 4 *Basic and Molecular Epidemiology of Gastrointestinal Disorders Research Center, Research Institute for Gastroenterology and Liver Diseases, Shahid Beheshti University of Medical Science, Tehran, Iran *

**Keywords:** Metabolic-associated fatty liver disease, Non-alcoholic fatty liver disease, Risk factors, Iranians

## Abstract

**Aim:**

This study aimed to identify the risk factors of metabolic (dysfunction)-associated fatty liver disease (MAFLD) among adults in northeastern Iran.

**Background:**

Non-alcoholic fatty liver disease (NAFLD) is the most common cause of chronic liver disease and hepatic manifestation of metabolic syndrome that threatens global public health. Recently, MAFLD has been proposed as a new terminology updated from NAFLD and diagnosed based on modified criteria.

**Methods:**

A nested case-control study was performed on the participants of the first phase of the Persian Sabzevar Cohort Study (PSCS), a survey that was conducted in northeastern Iran and enrolled 4,242 participants aged 35-70 years. In total, 968 MAFLD cases and 964 controls adjusted for age and sex were recruited. Data including demographic, lifestyle, anthropometric, biochemical, sleep pattern, and dietary intake information was collected.

**Results:**

The mean (SD [standard deviation]) age of participants was 49.2 (8.8) years, and 39.9% of the participants were males. The prevalence of MAFLD was 22.8% (95% CI [confidence interval] 19.2 – 26.3%). Increased body mass index (BMI) (OR [odds ratios] 5.51, 95% CI 2.73 – 11.10), waist circumference (WC) (OR 1.85, 95% CI 1.44 – 2.38), blood concentrations of triglycerides (TG) (OR 1.10, 95% CI 1.06 – 1.15), total cholesterol (TC) (OR 1.02, 95% CI 1.003 – 1.04), and alanine aminotransferase (AST) (OR 1.10, 95% CI 1.05 – 1.16) were significantly associated with an increased risk of the MAFLD (*p-*value <0.05). Furthermore, the odds of MAFLD risk was 43% higher in subjects who slept ≤ 5 hrs/day than those with ≥ 7 hrs per day of sleep (OR 1.43; 95% CI 1.07 – 1.92, *p-*value = 0.01).

**Conclusion:**

In this study, it was found that MAFLD was best predicted by BMI, WC, and serum levels of TG, total cholesterol, and AST. Sleeping ≤ 5hrs/day compared to ≥ 7hrs/day was associated with an increased risk of MAFLD.

## Introduction

 Non-alcoholic fatty liver disease (NAFLD) has become the most common etiology of chronic liver disease worldwide (1). In a recent meta-analysis of 86 studies from 22 countries, the global NAFLD prevalence was estimated to be approximately 25.2%, and the highest prevalence was reported in the Middle East (31.8%) and South America (30.4%); the lowest prevalence was found to be in Africa (13.5%) (2). NAFLD is a multi-stage disease, from simple steatosis to non-alcoholic steatohepatitis (NASH), which may progress to cirrhosis and hepatocellular carcinoma (3). It has been estimated that 15-20% of patients with NASH will have liver cirrhosis within 10-20 years (4). It is estimated that the number of liver-related deaths due to NASH will increase 178% by 2030 (5).

Previous evidence has shown that NAFLD is a multi-system disorder with extra-hepatic manifestations and is closely associated with metabolic disorders, including obesity, dyslipidemia, metabolic syndrome (MetS), type 2 diabetes mellitus (T2DM), chronic kidney disease (CKD), and cardiovascular disease (CVD)(6-12). A growing body of evidence suggests bidirectional associations between NAFLD and insulin resistance in metabolic disorders (13).

The prevalence rates of obesity, T2DM, and MetS among patients with NAFLD were 51%, 22.5%, and 42.5%, respectively (14). A panel of international experts from 22 countries proposed in 2020 that the name NAFLD be changed to metabolic-associated fatty liver disease (MAFLD) as a more appropriate term for the diagnosis of fatty liver disease (15). MAFLD is defined as the presence of hepatic steatosis in addition to having at least one of the metabolic features, such as overweight/obesity, T2DM, or metabolic dysfunction in lean subjects (16). In a recent meta-analysis study which included 2,667,052 participants in the general population, the prevalence of MAFLD was estimated to be 50.7% (46.9–54.4%) globally among overweight/obese subjects, with higher prevalence in males than females (17). 

Despite the alarming rate of increase in the global prevalence of NALD/MAFLD (18), there are no approved therapies for this condition, and lifestyle modifications remain as the most effective first-line treatment for MAFLD patients (19).

Genetic, environmental, and host risk factors and their interactions contribute to the incidence, progression, and severity of MAFLD. These potential risk factors may also contribute to the increased risk of MAFLD, depending on ethnicity, geographical location, demographics, and lifestyle-related parameters in various populations. To the best of the authors’ knowledge, this is the first study to investigate the determinants of MAFLD in Iranian adults. 

## Methods

***Study design and population***

A nested case-control study was conducted on participants of the first phase of the Persian Sabzevar Cohort Study (PSCS), which is a part of the PERSIAN cohort study. The PERSIAN (Prospective Epidemiology Research Studies in Iran) is a multicenter cohort study extending across 19 sites in different areas of Iran to identify the potential risk factors of common non-communicable diseases (NCD) among Iranian adults (20). The objectives, design, and characteristics of participants in the PSCS were previously published and are available at http://persiancohort.com. Briefly, 4,352 out of 5,174 eligible subjects aged 35–75 years who were residents of Sabzevar city were enrolled in the PSCS from 2014 to 2017.

In the current nested case-control study, cases were defined as subjects who were diagnosed at baseline with MAFLD, which was defined as having a fatty liver index (FLI) ≥60 (21) plus at least one of the following: being overweight or obese (body mass index [BMI] ≥25 kg/m^2^), having a T2DM diagnosis (defined as HbA1c ≥6.5% or being on any diabetes medication), or having any evidence of metabolic dysregulation. Metabolic dysregulation among individuals with hepatic steatosis and lean/normal weight who were not diagnosed with T2DM was defined as having at least two of the following metabolic risk abnormalities: 1) waist circumference ≥102 cm among men, and ≥88 cm among women; 2) blood pressure ≥130/85 mmHg or taking antihypertensive medication; 3) plasma triglycerides (TG) concentration ≥150 mg/dL or on a lipid-lowering medication; 4) plasma high density lipoprotein cholesterol (HDL-C) concentration <40 mg/dL among men and <50 mg/dL among women; 5) pre-diabetes (i.e. a fasting blood glucose (FBG) concentration 100 – 125 mg/dL, a 2-hour post-load glucose concentration 140 – 199 mg/dL, or a HbA1c 5.7% – 6.4%) (16). Controls were randomly selected from among the participants of the first phase of PSCS who had no liver disease with FLI<30. Cases and controls were matched for age and sex. 

**Figure 1:**
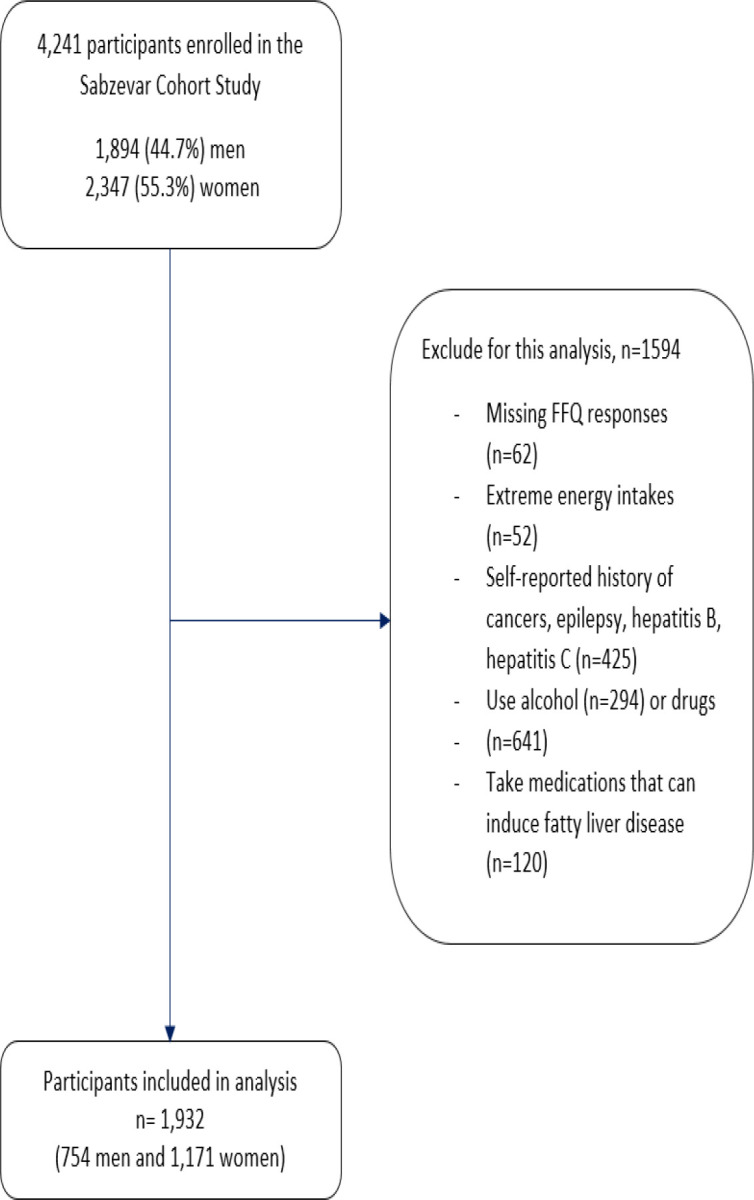
Participant flow chart

The study exclusion criteria were a history of hepatitis B or C, cancers, inflammatory bowel disease (IBD), celiac disease, Wilson disease, alcoholic fatty liver, organ transplantation, or the current use of alcohol or addictive drugs or medicine-induced fatty liver disease (valproate sodium, amiodarone, methyldopa, methotrexate, tamoxifen, naproxen, fluoxetine, nifedipine, gentamicin, tetracycline, and interferons). In addition, participants with more than 30 missing responses on the 116-item food frequency questionnaire (FFQ) (n=62), or those who had an average daily energy intake less than twice the value of the 25^th^ quartile or more than twice the value of the 75^th^ quartile (n=52) were excluded from the study. A total of 1,932 subjects (968 cases and 964 controls) were entered into the final analysis (Figure 1). 

***Data Collection***

The semi-structured questionnaire was used to collect data comprising socio-demographic characteristics, personal and family medical history, reproductive history, smoking and alcohol use, sleep habits, and the current use of medications and supplements. Dietary intake was assessed through a 116-item semi-quantitative food frequency questionnaire (FFQ) that was validated for the Iranian population (22). The International Physical Activity Questionnaire (IPAQ) Short Form, which was validated for the Iranian population, was used to assess physical activity status (23). Then, physical activity expenditure was calculated in metabolic equivalents of task-hours per day (MET-hours/day) and labeled as low (<0.59), moderate (0.59 – 0.67), or high (>0.67). Principal component analysis (PCA) was used to composite socioeconomic status (SES) using the variables of educational level, employment status, and household facilities (24).

***Anthropometrics***

To measure anthropometric indices, participants were asked to take off shoes and heavy clothes. Weight and height were measured using the calibrated SECA 755 scale (SECA, Hamburg, Germany) nearest to 1 kg and the SECA 204 stadiometer (SECA, Hamburg, Germany) nearest to 0.1 cm, respectively. Waist circumference (WC) was measured using a flexible measuring tape at midway between the lower rib margin and the iliac crest. Hip circumference (HC) was measured at the widest part of the hip. Body mass index (BMI) was calculated as weight (kg) divided by the square of height (m^2^). Waist-to-hip ratio (WHR) was calculated as waist circumference divided by hip circumference. 

***Blood pressure measurement***

After a 10-minute rest, a trained nurse measured systolic and diastolic blood pressure using a standard mercury sphygmomanometer (ALPK1, Japan) while participants were in a sitting position. For each participant, blood pressure was measured twice in the right and left arms at 5-minute intervals. Then, the four measurements were averaged to determine the systolic and diastolic blood for each participant. 

***Laboratory Measurements***

After 12–14 hours of overnight fasting, 10 ml of peripheral venous blood was obtained from each participant. Blood samples were subsequently centrifuged at 3000 rpm. Then, the derived serum and plasma were stored at -20 ℃ until used to measure fasting serum glucose (FPG), high-density lipoprotein cholesterol (HDL-C), triglyceride (TG), total cholesterol (TC), gamma-glutamyl transferase (GGT), alanine aminotransferase (ALT), and aspartate aminotransferase (AST). Assays were performed in the central laboratory of Sabzevar Medical University by commercial standard kits (Pars Azmoon Inc., Tehran, Iran) using an auto-analyzer (BT 1500, Biotecnica Instruments Spa, Rome, Italy). The level of LDL-C was evaluated using Friedwald formula in subjects with TG < 400 mg/dL and measured directly in others. FLI was calculated by the following formula (21): 

FLI = [e0.953×ln (TG) + 0.139×BMI + 0.718×ln (GGT) + 0.053×WC - 15.745 / (1 + e0.953×ln (TG) + 0.139×BMI +0.718×ln (GGT) + 0.053×WC - 15.745)] ×100.

***Metabolic Syndrome***

Two different criteria were used to diagnose MetS, namely the National Cholesterol Education Program’s Adult Treatment Panel III (NCEP/ATP-III) (25) and clinical diagnosis of MetS in Iranian adults (CCDMIA) (26). MetS was defined as the presence of at least three of the parameters presented in Table 1. 

***Ethics approval***

All participants signed a written informed consent form before enrolling in the study. The Ethics Committee of the Ministry of Health and Medical Education of Iran and Sabzevar University of Medical Science approved the PSCS study (approval number: IR.MEDSAB.REC.1398.137). The study protocol was approved by the Ethics Committee of Shahid Beheshti University of Medical Sciences (approval number: IR.SBMU.RIGLD.REC.1399.041). 

***Statistical Analysis***

Data was expressed as mean (SD) for continuous variables and as the number and percentage for categorical variables. The chi-square test was used for categorical variables and the student t-test was used for continuous variables to compare the two groups. Multivariable logistic regression was used to identify the MAFLD-associated risk factors. Covariates for the final model included age (years), sex, educational level (≤ high school/> high school), marital status, socioeconomic status (low/medium/high), and total energy intake (Kcal/day). All statistical analyses were performed using SPSS Version 22 (IBM Corp. Released 2013. IBM SPSS Statistics for Windows, Version 22.0 Armonk, NY: IBM Corp). A *p*-value of less than 0.05 was considered as statistically significant. 

**Table 1:** Two Criteria for clinical diagnosis of metabolic syndrome which was used in this study

Variables	NCEP/ATP	III CCDMIA
Abdominal obesity	WC>102 cm in men and>88 cm in women	WC≥95 in both sexes
Hypertriglyceridemia	TG≥150 mg/dL	TG ≥150 mg/dL
Low HDL-C level	<40 mg/dL in men and<50 mg/dL in women	<40 mg/dL in men and<50 mg/dL in women
Hypertension	SBP/DBP≥130/85 mmHg	SBP/DBP≥130/85 mmHg
Hyperglycemia	FBS≥110 mg/dL	FBS≥100 mg/dL

NCEP/ATP-III, National Cholesterol Education Program Adult Treatment Panel III; CCDMIA, Criteria for Clinical Diagnosis of metabolic syndrome in Iranian Adults; WC, Waist Circumference; TG, Triglyceride, SBP, Systolic Blood Pressure; DBP, Diastolic Blood Pressure; FBS, Fasting Blood Sugar.

## Results

A total of 1932 participants, 968 cases and 964 controls with a mean (SD) age of 49.2 (8.8) years and of whom 39.1% were males, were enrolled in this study. The baseline demographic and anthropometric characteristics of participants are presented in Table 2. The men to women ratios were 3:2 in both groups. Patients with MAFLD were more likely to be less academically educated, had low socioeconomic status and physical activity level, and had higher averages of weight, WC, WHR, BMI, SPB, and DBP relative to the controls. Most MAFLD patients were 40 – 60 years old (71.4%), female (60.1%), and had a BMI ≥30 kg/m^2^ (72.1%). Among the MAFLD patients, the percentage of women with high WC (≥88cm) was significantly higher than men (WC ≥102cm) (99.5% *vs.* 60.1%, *p*-value < 0.001). 

As shown in Table 3, MAFLD patients had higher serum levels of FBS, TG, TC, LDL-C, AT, AST, and GGT that were statistically significant and lower HDL-C than the controls. Additionally, low HDL-C (39% vs. 22%, *p*-value = 0.04) and high GGT (20.8% vs. 11.4%, *p*-value = 0.03) were more prevalent in women than men. 

MAFLD patients were categorized according to the concurrency of hepatic steatosis and one of the following metabolic abnormalities: overweight/obesity, T2DM, or metabolic dysfunctions. The data showed that patients who had both FLI>60 and overweight/obesity (BMI ≥ 25 kg/m^2^) had the highest proportion (84.7%) among MAFLD patients.

**Table 2:** Baseline demographic and anthropometric characteristics of MAFLD cases and controls in the PSCS (*n* = 1,932)

	MAFLD (n= 968)	non-MAFLD (n=964)	P-value*
Demographic variables			
Age (year) %(n)			0.31
<40	17.3 (167)	19.9 (192)	
40-60	71.4 (691)	68.9 (664)	
≥60	11.4 (110)	11.2 (108)	
Gender			0.26
Male %(n)	39.9 (386)	38.4 (370)	
Educational status %(n)			0.003
Illiterate	19.7 (145)	14.1 (104)	
Trade school	22.4 (165)	21.0 (155)	
Diploma	37.0 (273)	37.1 (274)	
University degrees	20.9 (154)	27.8 (205)	
Occupational status %(n)			0.14
Unemployed	3.5 (34)	2.7 (26)	
Employed	39.9 (383)	42.5 (407)	
Retired	8.7 (84)	10.8 (103)	
Housekeeper	47.9 (460)	44.1 (422)	
Socioeconomic status %(n)			0.01
Low level	37.3 (226)	30.3 (183)	
Moderate level	32.2 (197)	32.0 (193)	
High level	30.7 (187)	37.7 (228)	
Smoking, yes %(n)	7.1 (68)	7.7 (74)	0.31
Physical activity status %(n)			<0.001
Low level	39.9 (386)	25.1 (242)	
Moderate level	31.8 (308)	35.8 (345)	
High level	28.3 (274)	39.1 (377)	
Anthropometric variables			
Weight (kg)	84.79 ± 11.87	63.46 ± 8.92	<0.001
Waist circumference (cm) %(n)	105.05 ± 8.17	86.01 ± 7.27	<0.001
≥102 cm in men	60.1 (232)	0.3 (1)	<0.001
≥88 cm in female	99.5 (579)	39.4 (234)	<0.001
≥95 cm in Iranians in both gender	92.7 (897)	10.4 (100)	<0.001
BMI (kg/m2) %(n)	32.67 ± 4.23	24.40 ± 2.89	<0.001
<25	1.2 (11)	56.3 (515)	<0.001
25-30	26.7 (255)	40.9 (374)	
≥30	72.1 (689)	2.8 (26)	
WHR	0.93 ± 0.06	0.87 ± 0.06	<0.001
SBP (mmHg) ≥130, %(n)	22.7 (218)	7.6 (72)	<0.001
DBP (mmHg) ≥85, %(n)	11.9 (114)	3.3 (31)	<0.001

Abbreviations: BMI, body mass index; DBP, diastolic blood pressure; MAFLD, metabolic associated fatty liver disease; PSCS, Persian Sabzevar Cohort Study; SBP, systolic blood pressure; WHR, waist-to-hip ratio; * form student t-test for continuous variables and the chi-square test for categorical variables

The current data further showed that MAFLD patients were more likely to have hypertension (70.7% vs. 29.3%, ischemic heart disease (57.1% vs. 42.9%), and MetS (94.0% vs. 6.0%) relative to the controls (Table 4). Dietary habits and sleep duration were not statistically different between two groups (Table 4). 

As presented in Table 5, BMI (OR 5.51, 95% CI 2.73 – 11.10), WC (OR 1.85, 95% CI 1.44 – 2.38), serum levels of TG (OR 1.10, 95% CI 1.06 – 1.15), TC (OR 1.02, 95% CI 1.003 – 1.04), and AST (OR 1.10, 95% CI: 1.05 – 1.16) could predict the MAFLD risk (NagelkerkeR^2 ^= 0.98). Furthermore, the odds of MALFD were significantly 2.21 times higher in subjects who had sedentary lifestyles than those with high physical activity (OR 2.21; 95% CI 1.63 – 2.99, *p*-value < 0.001). Subjects who consumed ≤3 meals per day had 44% higher (OR 1.44; 95% CI 1.08 – 3.42, *p*-value = 0.04) MAFLD risk than those with ≥ 4 meals per day. The results also indicated that subjects who slept less than 5 hours/day had a higher risk of MAFLD than those sleeping more than 7 (OR 1.43; 95% CI 1.07 – 1.92, *p*-value = 0.01).

**Table 3:** Baseline biochemical characteristics of MAFLD cases and controls in the PSCS (*n* = 1,932)

Biochemical variables	MAFLD(n= 968)	non-MAFLD (n=964)	P-value*
FBS (mg/dL)	116.20 ± 44.67	99.14 ± 34.21	<0.001
High FBS,%(n) (≥100 mg/dL)	54.4% (527)	24.5% (236)	<0.001
TG (mg/dL)	200.48 ± 139.69	94.30 ± 40.03	<0.001
High TG, %(n)(≥ 150 mg/dL)	58.8 % (569)	9.1% (88)	<0.001
TC (mg/dL)	202.20 ± 41.33	182.00 ± 36.14	<0.001
High TC, %(n) (≥ 200 mg/dL)	49.9 (483)	30.3% (292)	<0.001
HDL-C (mg/dL)	50.47 ± 10.29	55.72 ± 10.47	<0.001
Low HDL-C, %(n) (< 40 mg/dL in men)	22.0% (85)	10.3% (38)	<0.001
Low HDL-C, %(n) (<50 mg/dL in women)	39.0% (227)	21.4% (127)	<0.001
LDL-C (mg/dL)	112.97 ± 34.91	107.86 ± 31.21	0.001
High LDL-C, %(n) (≥ 130 mg/dL)	30.7% (296)	22.5% (217)	<0.001
ALT(IU/L)	26.44 ± 18.05	17.66 ± 10.80	<0.001
High ALT, %(n) (≥40 IU/L)	12.6% (122)	2.5% (24)	<0.001
AST(IU/L)	21.72 ± 12.62	18.63 ± 6.05	<0.001
High AST, %(n) (≥40 IU/L)	3.9% (38)	0.7% (7)	<0.001
GGT(IU/L)	34.39 ± 32.82	17.20 ± 8.86	<0.001
High GGT, %(n) (> 61 IU/L in men)	11.4% (44)	0.5% (2)	<0.001
High GGT, %(n) (> 36 IU/L in women)	20.8% (121)	1.7% (10)	<0.001
ALP (IU/L)	236.96 ± 72.92	203.85 ± 56.99	<0.001

Abbreviations: ALT, alanine aminotransferase; AST, Aspartate aminotransferase; FBS, Fasting blood sugar; GGT, gamma-glutamyl transferase; HDL-C, High-density lipoprotein cholesterol; LDL-C, Low-density lipoprotein cholesterol; MAFLD, Metabolic associated fatty liver disease; PSCS, Persian Sabzevar Cohort Study; TC, Total cholesterol; TG, Triglyceride; * form student t-test for continuous variables and the chi-square test for categorical variables

**Table 4:** Baseline co-morbidities and dietary habits of MAFLD cases and controls in the PSCS (*n* = 1,932)

	Total^1^	MAFLD (n= 968)	non-MAFLD(n=964)	P-value*
Co-morbidities				
Overweight/ obesity(BMI ≥ 23kg/m^2^)	84.7% (1637)	59.1% (967)	40.9% (670)	<0.001
Diabetes	12.3% (238)	71.8% (171)	28.2% (67)	<0.001
Normal weight with metabolic abnormalities	12.3% (1380)	68.2% (941)	31.8% (439)	<0.001
Hypertension	23.0% (441)	70.7% (312)	29.3% (129)	<0.001
Ischemic heart disease	8.8% (168)	57.1% (96)	42.9% (72)	<0.03
Stroke	1.0% (19)	47.4% (9)	52.6% (10)	0.49
Thyroid disorders	10.0% (192)	53.1% (102)	46.9% (90)	0.21
Metabolic syndrome (NCEP/ATPIII)	24.9% (482)	94.0% (453)	6.0% (29)	<0.001
Dietary Habits				
Number of meals per day				0.33
<3 meals/day	18.0% (347)	53.0% (184)	47.0% (163)	
3 meals/day	81% (1564)	49.4% (773)	50.6% (791)	
≥4 meals/day	1% (17)	52.9% (9)	47.1% (8)	
Frequency of eating fried foods				0.13
1-3 times/month	1.8% (32)	65.6% (21)	34.4% (11)	
1-3 times/week	18.2% (332)	52.1% (173)	47.9 % (159)	
daily	80.1% (1462)	48.3% (721)	50.7% (741)	
The way of storing vegetables				0.17
Raw	32.5% (595)	48.6% (290)	51.3% (305)	
Boiled	4.5% (82)	3.1% (56)	4.5% (26)	
Fried	63.0% (1152)	49.9% (575)	50.1% (577)	
Sleep duration (hours/day)		7.20 ± 1.45	7.19 ± 1.41	0.56

Abbreviations: BMI, body mass index; MAFLD, metabolic associated fatty liver disease; NCEP/ATPIII, National Cholesterol Education Program’s Adult Treatment Panel III (NCEP/ATP-III); PSCS, Persian Sabzevar Cohort Study; ^1^Expressed as percentage of total population (number); ^2^ Expressed as percentage of population with that conditions (number); * form student t-test for continuous variables and the chi-square test for categorical variables.

**Table 5:** Predictive risk factors of MAFLD in the PSCS (*n* = 1,932)

Variables	OR*	SE	95% CI	P-value
BMI (kg/m^2^)	5.51	0.357	2.73 – 11.10	<0.001
WC (cm)	1.85	0.128	1.44 – 2.38	<0.001
TG (mg/dL)	1.10	0.020	1.06 – 1.15	<0.001
Cholesterol (mg/dL)	1.02	0.011	1.00 – 1.04	0.02
AST (IU/L)	1.10	0.025	1.05 – 1.16	<0.001
Number of meals per day				
< 3 meals/day	1.44	0.33	1.08 – 3.42	0.04
3 meals/day	1.12	0.32	0.22 – 2.67	0.88
4-5 meals/day	Ref (1)			
Sleep duration				
< 5 hr/day	1.43	0.15	1.07 – 1.92	0.01
5-6 hr/day	1.21	0.17	0.86 – 1.70	0.26
6-7 hr/day	1.10	0.20	0.74 – 1.64	0.60
> 7 hr/day	Ref (1)			
Physical activity level				
Low	2.21	0.15	1.63 – 2.99	< 0.001
Medium	1.20	0.14	0.89 – 1.61	0.21
High	Ref (1)			

Abbreviations; AST, Aspartate aminotransferase; BMI, Body Mass Index; MAFLD, Metabolic associated fatty liver disease; OR, Odds Ratio; PSCS, Persian Sabzevar Cohort Study; SE, Standard Error; TG, Triglyceride; WC, Waist Circumference; * From unconditional logistic regression models

## Discussion

To the best of the authors’ knowledge, this is the first study to assess the prevalence and risk factors of MAFLD in the community population of Northeastern Iran using the new definition of MAFLD. It was found that the overall prevalence of MAFLD in the study population was 22.8%, which is consistent with the 29.2% prevalence of MAFLD observed in a study conducted by Fan et al. among 5,377 participants aged 30-79 years recruited from South China (27). In 2021, the global prevalence of MAFLD among overweight or obese individuals in the general population was estimated to be 50.7% (95% CI 46.9–54.4), based on 1,925,147 subjects (2). The MAFLD prevalence was higher in men (59%) than in women (47.5%). The findings of this meta-analysis showed that the prevalence of MAFLD was 52.1% among 2,565,388 participants from 57 studies (2). In the present study, the prevalence of MAFLD was approximately 2 times higher in women than in men (60.1% vs. 39.9%). Consistent with the current findings, Fan et al. reported a higher prevalence of MAFLD in men than in women (31.7% vs. 25.5%) (27). However, the mean age of participants in the study was 67 years, which was older than the mean age of 49 years in the current study. The present study also found that MAFLD patients who concurrently had hepatic steatosis and overweight/obesity accounted for a higher portion (84.7%) of MAFLD patients. This finding is consistent with the study of Fan et al. (27), who reported that overweight/obesity accounts for 90.5% of MAFLD patients. The current results revealed that participants with obesity had 5.51-times ORs of MAFLD than normal weight participants. By considering this point, the higher prevalence of overweight/obesity in women relative to men in the current study can explain the higher rate of MAFLD among women than men. The findings further showed that MAFLD patients had a significantly higher waist circumference in both sexes and a higher portion of obesity (BMI≥30 kg/m^2^) compared to the controls, which may indicate the higher prevalence of abdominal obesity in MAFLD patients. It is well known that abdominal obesity is one of the most important risk factors for both progression and severity of chronic diseases, including fatty liver disease (28). The current findings are consistent with previous studies suggesting that overweight/obesity can have a significant effect on MAFLD. The present study also showed that the proportion of abnormal metabolic features was significantly higher in patients than in those without MAFLD. Many previous studies have confirmed the close associations of NAFLD with T2DM and MetS components. It has been demonstrated that insulin resistance can play a central role in the vicious circle between hepatic steatosis in NAFLD/MAFLD and metabolic disorders (13). From the mechanistic perspective, previous studies have suggested that hepatocytes act as an endocrine organ, which secrets a wide array of hepatokines, protein and lipid metabolites, and non-coding RNAs (miRNAs). These liver-derived factors serve as autocrine/paracrine or endocrine mediators that strongly affect metabolic pathways, such as insulin signaling in the liver and peripheral tissues (29).

In this study, logistic regression revealed that higher levels of TG, TC, and AST were associated with a higher OR of NAFLD in the general population. Li et al. conducted a population-based cross-sectional study on 1,608 cases with normal ALT categorized as obese and non-obese patients and found that TG was associated with a 2.54-fold increase in NAFLD among non-obese subjects (30). They suggested that BMI and TG should be considered to prevent NAFLD, especially in patients with normal weight (30). Results of a review performed by Deprince et al. (2020) also showed that alterations in hepatic lipid and lipoprotein metabolism are the main factors affecting an increased risk of cardiovascular disease (CVD) in NAFLD patients (31). It was shown that CVD is the leading cause of complications and death in NAFLD patients. The close associations between CVD and NAFLD emphasize the need for early identification and treatment of CVD risk factors in patients with NAFLD (32). Lee et al. conducted a nationwide cohort study on 9,584,399 Korean individuals aged 40-64 years during 10 years of follow-up and reported that the change in diagnosis criteria from NAFLD to MAFLD might identify a greater number of patients who were at increased risk of CVD (33).

In this study, it was observed that participants with a daily intake of food less than 3 meals per day were 1.44 times more likely to have MAFLD compared to subjects who ate ≥ 4 meals per day. Similarly, Travato et al. conducted a cohort study on 708 non-diabetic participants aged 15-35 years and reported the sleep shortage and smaller number of meals frequency in NAFLD patients relative to non-NAFLD subjects (34). Recently, in a prospective cohort study performed on 8,874 community dwelling Chinese people aged over 45 years with 4 years follow-up, Wang et al. reported that eating four compared to three meals per day was significantly associated with a lower incidence of T2DM, especially in normal weight subjects (35). 

Furthermore, the current study found that MAFLD risk was 43% higher in those who slept less than 5 hours per day than those with more than 7 hours/day of sleep. There is an inconsistency in relationship between sleep duration and the risk of NAFLD/MAFLD. Another study from a historical cohort of 12,306 participants in Japan found that sleep duration of less than 5 hours/day may increase the risk of NAFLD in both genders compared to sleeping more than 7 hours/day in men (HR 1.39, 95% CI 1.13 – 1.72, *p*-value = 0.002) and in women (HR 1.46, CI 1.05 – 2.04, *p*-value = 0.02) (36). Moreover, another study from Korea reported a significant relationship between long sleep duration and an increased risk of NAFLD among the middle-aged population who participated in the Korean Genome and Epidemiology study (37). Several plausible mechanisms have been suggested to explain the association between sleep and NAFLD. In one mechanism, it is described that sleep deprivation was associated with increasing ghrelin and decreasing leptin levels, which increase appetite and weight gain (38). It has been suggested that inadequate sleep duration and poor sleep quality may increase the risk of insulin resistance and impaired glucose metabolism (39, 40). Another mechanism is supposed to be related to increasing the secretion or activity of pro-inflammatory mediators such as interleukin 1 (IL-1) or tumor necrosis factor-alpha (TNF-α) in sleep deprivation, which play a crucial role in the development of NAFLD (41). 

The current study had several strengths and limitations. One of strengths of this study was the investigation of the prevalence and potential risk factors of MAFLD for the first time among a sample of the Iranian population. In addition, this study was conducted with a relatively large sample size as a community-based study with comprehensive clinical, anthropometric, and dietary habits/lifestyle parameters, which cover the various aspects of the common lifestyles of the studied population. 

Some limitations were also encountered. The main limitation was the cross-sectional nature of this study, which limited analysis to the inferential type. Therefore, the authors have planned a longitudinal study to be performed after the next phase of PSCS. Another limitation was that this study was conducted in the Sabzevar area of Iran, which may limit the generalization of the findings to other populations. Finally, no ultrasonography or liver biopsies were performed to assess fatty liver disease. Therefore, there is no data about the grade and severity of NAFLD in the current patients. However, the validity of FLI relative to liver biopsy as a gold standard of diagnosing NAFLD was confirmed in previous studies, and the use of FLI may decrease the accuracy of NAFLD diagnosis. 

The current results suggest that MAFLD was best predicted by the general risk factors such as BMI, WC, TG, cholesterol, and AST in the general population. In addition, among lifestyle variables, lower education level, sedentary lifestyle, and short sleep duration showed significant associations with increased odds of MAFLD.

## Conflict of interests

The authors declare that they have no conflict of interest.
